# Supplementary material for: Prevention and management of anaemia in pregnancy: Community perceptions and facility readiness in Ghana and Uganda
Source: PLOS Glob Public Health. 2024 Aug 26;4(8):e0003610. doi: 10.1371/journal.pgph.0003610 (PMC11346734; doi:10.1371/journal.pgph.0003610)
Supplement: S1 Data — (PDF) [file pgph.0003610.s002.pdf]

Health Facility Assessment: Maternal Anaemia

**Facility Information**			
**1**	Facility name and number		[Enter text]
**2**	Your name		[Enter text]
**3**	Name of facility in-charge		[Enter text]
**4**	Contact details of facility in-charge		[Enter text]
**5**	Date(s) of facility assessment		[Enter text]

**Module E. Anaemia<br>To be carried out with: Clinical maternity staff member**			
	**Question**	**Instruction**	**Response**
**1**	Are there standards/ processes/ guidelines for *anaemia identification* at this hospital?	Ask to see standards	Available and seen<br>Not seen (reported available)<br>Not available<br>Unsure/don't know
**2**	Which haemoglobin tests are available at this hospital?	Ask to see test, select all responses that apply	Automated analyser<br>Manual test<br>Other [insert text]
**3**	Are there point-of-care haemoglobin tests at this hospital?		Yes<br>No<br>Unsure/don't know
**4**	Does this hospital *routinely* give pregnant women iron and folic acid tablets?		Yes, all pregnant women<br>Only prescribed to women living in higher-risk areas<br>No, this is not given routinely
**5**	Does this hospital *routinely* give pregnant women IPT for malaria?		Yes, all pregnant women<br>Only prescribed to women living in higher-risk areas<br>No, this is not given routinely
**6**	Does this hospital *routinely* give pregnant women deworming tablets?		Yes, all pregnant women<br>Only prescribed to women living in higher-risk areas<br>No, this is not given routinely
**7**	Number of women presenting during labour in the past three months	Maternity register review	[Number]
**8**	Number of women presenting during labour in the past three months who were tested for anaemia	Maternity register review	[Number]

Focus Group Discussion Guide: Maternal Anaemia

Participants:

- Women (pregnant or recently delivered)
- Male partners of pregnant or recently delivered women
- Community influencers (leaders, village elders)
- Blood donors

**Antenatal Care**	**Responses**
1. How far along in their pregnancies are women in this community when they usually go for antenatal care?	
2. What happens during antenatal care? (*Probe: what services do women receive? What sorts of problems might be detected in antenatal care?)*	
3. Where do most women in this community go for antenatal care for their early (first and second time) appointments?<br>a. Do you know how many times a woman should go for antenatal care throughout her pregnancy?	
4. Is it important for pregnant women to attend antenatal care? (Please explain)	
5. Do women face any barriers in trying to go for antenatal care? (Please explain)<br>a. Are women expected to pay for anything in antenatal care? (Please explain)	
6. Are pregnant women in this community satisfied with their antenatal care? (Please explain)	
**Anaemia**	
1. What is anaemia?	
2. What does anaemia cause?<br>a. For pregnant women?<br>b. For babies?	

3. How would you know if you had anaemia? (Please explain) (*Probe: what is the role of antenatal care in diagnosing anaemia? Are you aware of any blood tests to diagnose anaemia?*)	
4. Do you know how anaemia is treated? (Please explain) (*Probe: what is the role of antenatal care in treating anaemia?)*<br>a. Do you know why people are given iron and folic acid during pregnancy?<br>b. Do you know why people are sometimes treated for malaria during pregnancy?<br>c. Do you know why people are sometimes treated for worms during pregnancy?	
5. Is there anything that a pregnant woman can personally do to try and prevent or treat anaemia?	

In-depth Interview Guide: Maternal Anaemia

Participants:

- Maternity staff: attending physicians, midwives/nurses and clinical assistants
- Senior management of maternity staff

**Question**	**Responses**
1. Briefly describe the referral system in place at this hospital (to-and-from the facility) (*Probe: anything specific for severe anaemia? How do facilities communicate with one another?)*	
2. How is it determined that a woman with maternal bleeding (antenatal or postpartum haemorrhage) needs a blood transfusion? (*Probe for: clinical indicators, anaemia severity, blood loss*)	
3. Is anaemia measured in this hospital for women coming for care during labour and childbirth? (Please explain how)	
4. How is maternal anaemia prevented or managed at this facility?	

<u>Key Informant Interviews: Maternal Anaemia</u>

<u>Participants:</u>

Ghana

- District Director of Health Services/their deputies
- Maternal and Child Health Focal Person/their deputies

Uganda

- District Health Officers/their deputies
- In-charges of Reproductive Health/their deputies
- Procurement Officers for reproductive and child health
- District Health Educator

**Questions**	**Responses**
1. How are staff trained to diagnose anaemia during antenatal care? *(Probes: using laboratory tests? Using point-of-care tests?)*<br>a. If clinical assessment is indicated: do you feel this approach is accurate?	
2. How are staff trained to treat anaemia during antenatal care?	
3. What are the main barriers to the detection and management of anaemia during pregnancy?	
4. How are health facility staff trained to detect anaemia at the time of labour and childbirth?	
5. What are the main barriers to anaemia detection at the time of labour and childbirth?	
6. How are staff trained to manage anaemia at the time of labour and childbirth?	
7. What are the main barriers to the management of anaemia during labour, delivery, and immediately post-partum?	
