# Supplementary material for: Prevention and management of anaemia in pregnancy: Community perceptions and facility readiness in Ghana and Uganda
Source: PLOS Glob Public Health. 2024 Aug 26;4(8):e0003610. doi: 10.1371/journal.pgph.0003610 (PMC11346734; doi:10.1371/journal.pgph.0003610)
Supplement: Attachment — Submitted filename: Comments to Authors.docx [file pgph.0003610.s003.docx]

**Comments to Authors**

The manuscript is well-written and addresses an important issue prevalent in low-resource settings. It also contributes to the body of literature on the subject.

Kindly address the following:

1. The citations should be inserted before punctuation marks.
2. The methods section should include more details on the sample size for the FDGs, IDIs, and KIIs.
3. Line 119: The Methods section should have a heading for “quantitative data” and “qualitative data.”
4. Lines 128-132: Please provide a citation for this methodology if available.
5. Line 158: how were the IDIs, FGS, and KIIS interviews recorded? Written or audio recording? Also consider adding the sample sizes for each of the IDIs, FGS, and KIIS. E.g IDIs (n=x)
6. Line 167: What are these tools and what do they measure?
7. This statement is confusing. “Since none of the tools needed minimal revision following piloting, we included data from the pilots in our analysis.” Did you mean the tools needed minimal revision?
8. Line 181: Consider adding the word “**Quantitative”** to the phrase “data from the health facility assessments..”
9. Table 1: Add a row for “Total” for each district and Country separately.
10. Table 2: consider adding demographic information if available (Gender and Age)
11. The results section should also include a table for the qualitative findings on the “availability of appropriate standards or protocols, drugs, equipment, trained staff, and infrastructure to support anaemia prevention and management”, “measurement of haemoglobin at the time of labour” as stated in the methodology. This should include the country, data sources and findings.
12. Consider: The result section should separate qualitative findings from quantitative findings. The findings should be reported under what the tools were measuring (especially for the quantitative findings).
13. Line 267: the statement should be restructured. Human error and equipment error are two different things.
14. Lines 313-315: Is this a statement from multiple sources? I see (FGD, community influencers, Uganda). Please clarify- This format is also repeated many times in the manuscript.
15. Lines 349-359 appear to fit under different topics (consider a new heading for it)
16. Line 370: What type of program? Food and nutrition education programs?
17. Line 423: Should this be italicized? It appears to be a subheading under “ANC and the prevention and management of anaemia in pregnancy”
18. Lines 427-429: What was the average number or range of ANC visits mentioned by respondents?
19. Lines 531-532 are confusing. Please restructure.
20. Lines 534-537: Is this by women in the FGDs or the Community influencers? The succeeding quotation in lines 539 to 541 is by multiple sources (refer to comment 15 above).
21. Lines 611-616: this should also be stated earlier under the results from quantitative findings
22. Line 645: consider restructuring to “This mixed methods assessment explored facility readiness and diverse stakeholder…”
23. Lines 679-681: Add some sentences on the value of this for reducing anaemia among pregnant women. This knowledge can be further emphasized during ANC.
24. The discussion should also discuss financial barriers to accessing care during ANC as cited by the respondents.
25. Line 729: Limitations should include geographical limitations of the study sites especially for generalization purposes. The findings would only apply to similar facilities and populations in sub-Saharan Africa.
