# Supplementary material for: Prevention and management of anaemia in pregnancy: Community perceptions and facility readiness in Ghana and Uganda
Source: PLOS Glob Public Health. 2024 Aug 26;4(8):e0003610. doi: 10.1371/journal.pgph.0003610 (PMC11346734; doi:10.1371/journal.pgph.0003610)
Supplement: Attachment — Submitted filename: Anaemia paper response to reviewers.docx [file pgph.0003610.s004.docx]

We thank the editor and the reviewers for their really useful comments on the manuscript. We have made revisions that we feel have strengthened the quality of the manuscript.

**Editor comments**

Concerning the methods, please see the comments from the reviewers requesting additional information. I would also encourage the authors to review COREQ or similar reporting guidelines to add additional information concerning the qualitative methods.

**We have added further details as per the reviewers’ requests and some additional detail to ensure clarity in our methods.**

The quantitative methods could also use a little more description, in terms of why the sample size was chosen, and why further analyses were not conducted with the data.

**Apologies that this was not quite clear. There was no sample size calculation, as we simply went with a census—all facilities meeting our inclusion criteria were included. Further analyses were not conducted because this was not the intention of the study, nor was it powered to do so. We have added in some further details so that this is clear.**

I also encourage the authors to publish their data collection instruments and final qualitative codebook as appendices/supplemental materials.

**We have uploaded our data collection instruments as supplemental files.**

The choice of Ghana and Uganda for the work needs to be explained--I understand that this was a sub-study, but it still isn't clear why these two countries were chosen.

**Thank you, we have added why we have chosen these countries.**

Finally, the authors need to explain how this is a mixed methods study. The type of mixed methods design should be named, and the point(s) of integration of qual and quant methods/data needs to be explained. As is, this seems more like a multi-method study, without integration between quant and qual findings, rather than a mixed methods study.

**We aimed to present findings together, with the qualitative data shedding insight on some of the findings from the quantitative survey. The instruments were designed to be complementary so that the qualitative findings could shed some light on the quantitative findings (and have now made this clear in our methods). For instance, we noted from patient records in the survey if haemoglobin was measured at the time of labour, then directly asked staff about this during interviews. As such, we believe we utilised the different data collection approaches together and presented a synthesis across both rather than quantitative findings alone and qualitative findings alone. There are of course some instances were data were only captured quantitatively or qualitatively, however, we do not believe that this would discredit the suggestion that this was mixed methods.**

Consider expanding the limitations concerning the quantitative data, if a power/sample size analysis was not the basis for the quantitative data collection, which may in turn have limited the analyses possible.

**Thank you for this comment. However, we don’t actually see this as a limitation as it was never a goal of this research to do further statistical analysis. It was very much intended to be descriptive as the state of these types of services is simply not well documented. We did add (as per reviewer 1’s comment) a line about how our results are not generalizable.**

**Reviewer #1 comments**

The manuscript is well-written and addresses an important issue prevalent in low-resource settings. It also contributes to the body of literature on the subject.

**Thank you very much.**

Kindly address the following:

1. The citations should be inserted before punctuation marks.

**Within Vancouver referencing style, convention is typically that the reference is placed outside of punctuation. Please see various guidance which reiterate this point (**[**https://guides.library.uq.edu.au/referencing/vancouver/in-text-citations#:~:text=The%20placement%20of%20citation%20numbers,outside%20full%20stops%20and%20commas**](https://guides.library.uq.edu.au/referencing/vancouver/in-text-citations#:~:text=The%20placement%20of%20citation%20numbers,outside%20full%20stops%20and%20commas)**,** [**https://subjects.library.manchester.ac.uk/referencing/referencing-vancouver**](https://subjects.library.manchester.ac.uk/referencing/referencing-vancouver) **). However, some journals prefer it within, regardless, so if the editorial team would prefer that the reference is inside the punctuation we can revise this.**

2. The methods section should include more details on the sample size for the FDGs, IDIs, and KIIs.

**The IDIs follow specific personnel in each participating health facility, there was no predetermined sample size—we sampled those members of staff with the relevant insights to be able to fully answer our questions who were available. For KIIs, again, we sampled all people occupying specific roles and by nature of being key informants, there is no pool to sample from. Finally, for the FGDs, we wanted a range of views and divided groups according to role in the community and age to enable the full participation of participants—we aimed for 6–10 people in each FGD. We have added some text to make this more clear.**

3. Line 119: The Methods section should have a heading for “quantitative data” and “qualitative data.”

**Thank you, this has been added.**

4. Lines 128-132: Please provide a citation for this methodology if available.

**This is not a specific methodology, simply capture of information from patient files, and we unfortunately could not find a suitable reference.**

5. Line 158: how were the IDIs, FGS, and KIIS interviews recorded? Written or audio recording? Also consider adding the sample sizes for each of the IDIs, FGS, and KIIS. E.g IDIs (n=x)

**Thank you, we have noted that these were audio recorded. The sample sizes are noted in Table 2.**

6. Line 167: What are these tools and what do they measure?

**Apologies for the confusion. “Tools” are our data collection instruments. We’ve revised this to be more clear.**

7. This statement is confusing. “Since none of the tools needed minimal revision following piloting, we included data from the pilots in our analysis.” Did you mean the tools needed minimal revision?

**Agreed, we have revised this wording.**

8. Line 181: Consider adding the word “**Quantitative”** to the phrase “data from the health facility assessments..”

**Agreed, we have added this.**

9. Table 1: Add a row for “Total” for each district and Country separately.

**Thank you for the comment. We intentionally did not add totals. These details are to give an overview of the sites from which data were collected on an individual basis and not to present a district- or country-level snapshot. We feel that presenting totals would be confusing to the reader as we did not, for instance, sample across all clients—we are simply highlighting the range in client volumes across the sites we did include.**

10. Table 2: consider adding demographic information if available (Gender and Age)

**Unfortunately these data are not available for all participants.**

11. The results section should also include a table for the qualitative findings on the “availability of appropriate standards or protocols, drugs, equipment, trained staff, and infrastructure to support anaemia prevention and management”, “measurement of haemoglobin at the time of labour” as stated in the methodology. This should include the country, data sources and findings.

**Thank you for this comment. Assuming that it is the *quantitative* (not qualitative) findings being referred to, we have added Table 3 in our results.**

12. Consider: The result section should separate qualitative findings from quantitative findings. The findings should be reported under what the tools were measuring (especially for the quantitative findings).

**As we used mixed methods and intentionally had our quantitative and qualitative instruments complement one another, we would prefer to keep our results presented as they are.**

13. Line 267: the statement should be restructured. Human error and equipment error are two different things.

**We agree this is confusing. It’s both, so we’ve rephrased to make this clear.**

14. Lines 313-315: Is this a statement from multiple sources? I see (FGD, community influencers, Uganda). Please clarify- This format is also repeated many times in the manuscript.

**In our methods we note that “community influencers” refers to “community leaders and elders (“community influencers”)”. When we refer to findings from those FGDs—where this is stated it is the FGDs carried out with community leaders and elders.**

15. Lines 349-359 appear to fit under different topics (consider a new heading for it)

**Thank you for this comment. As authors, we reflected on this point but agree it fits where it is because the description of what is being tested is still linked to understanding of symptoms (“volume of blood”—or doing a clinical assessment by looking at the hands to “see if you have blood”—again, the link to pallor and understanding of symptoms).**

16. Line 370: What type of program? Food and nutrition education programs?

**Apologies if this isn’t clear, but it is immediately in the line that follows (pregnancy schools and community-based nutrition programs).**

17. Line 423: Should this be italicized? It appears to be a subheading under “ANC and the prevention and management of anaemia in pregnancy”

**Yes, thank you. We have italicized this.**

18. Lines 427-429: What was the average number or range of ANC visits mentioned by respondents?

**We did not provide this value as it was not asked consistently across each participant. The reason we have the value on initiation of ANC timing in Ghana is because this was asked of every woman in the FGD by the facilitator, but this was not the case in Uganda, nor for the other participant types.**

19. Lines 531-532 are confusing. Please restructure.

**Apologies, part of this sentence belonged in the preceding one and we have adjusted.**

20. Lines 534-537: Is this by women in the FGDs or the Community influencers? The succeeding quotation in lines 539 to 541 is by multiple sources (refer to comment 15 above).

**Apologies for the confusion. This was stated by women (“Some women stated…”). Throughout, we only refer to “women” to be pregnant or recently delivered women, and “community influencers” to refer to the leaders/elders that make up those FGDs.**

21. Lines 611-616: this should also be stated earlier under the results from quantitative findings

**Our results from quantitative findings are not all in one place and we feel they sit better under this heading.**

22. Line 645: consider restructuring to “This mixed methods assessment explored facility readiness and diverse stakeholder…”

**Agreed, we have revised the line.**

23. Lines 679-681: Add some sentences on the value of this for reducing anaemia among pregnant women. This knowledge can be further emphasized during ANC.

**Thank you for this comment, we have added a sentence on this and commented that this is already well-discussed in ANC.**

24. The discussion should also discuss financial barriers to accessing care during ANC as cited by the respondents.

**In the original text (line 689) we provided a citation that notes financial barriers in both contexts prohibit uptake of ANC. In lines 697–701 we note, “In both countries, there were common barriers around ANC uptake, largely stemming from long distances to the health facilities, difficulty finding or paying for transportation, other costs, especially for medications, and poor staff attitudes” and provide a reference that this resonates with other contexts (line 702).**

25. Line 729: Limitations should include geographical limitations of the study sites especially for generalization purposes. The findings would only apply to similar facilities and populations in sub-Saharan Africa.

**Thank you, we have added a line to this effect.**

**Reviewer #2 comments**

Thank you for sharing your work with us. It was an eye-opening experience reading your work. However, there are several points I would like to share.

**Great, thank you very much.**

1. First, related to the references, some of your references are outdated (older than 5 years) and irrelevant to the sentence prior to the citation. When you put a
   citation behind a sentence, the citation must justify the sentence.

**All of our references were considered for their relevance. We do not provide, for instance, prevalence estimates that are dated, as we agree the timeliness of such a reference would be problematic. However, the authors have reviewed are reference list thoroughly and agree with the relevance of the papers cited here.**

2. Second, The use of the journal's template is advisable to improve the flow and readability of the manuscript. Follow the template as closely as possible.

**Apologies, we are not sure what this is referring to as there is no specific journal template for PLoS Global Health, barring the overarching headings which we have followed. This is also checked by the editorial team at the time of submission and the authors were not alerted to any issues. We are open to revising the formatting as necessary should this be identified.**

3. Ensure that the presentation of tables comply with the journal's style and formatting guidelines.

**Again, we are unsure of what the issue is as the tables are formatting as per the journal’s guidelines and no issues were flagged by the editorial team.**

4. Discussion of research results: The discussion should provide arguments for the research results that have been claimed, have a logical cause-and-effect explanation and be structured in the form of a 'new story'.

**Thank you for your comment. There are many considerations for a discussion, but we do not believe a descriptive assessment that presents a snapshot of facility readiness and community understanding of anaemia warrants a “cause-and-effect” explanation. We have not heard of a discussion needing to be structured as a “new story”, though have taken efforts to detail what our study contributes to literature.**

5. References are advisably from reputable journal articles.

**We are unsure if the reviewer is requesting that we need more reputable journals or that our references are from reputable journals? Please see our response to comment 1 about selection of references included.**

6. Local references should be kept to a maximum of 10% from total references. Many of the references are not recent.

**We are not sure where this particular guideline is coming from as local references are often incredibly important to contextualize and interpret findings and we have never seen a 10% cutoff stated, certainly not by the journal. This issue was not picked up by the editorial team.**

7. There enough is not detail in order to replicate the study.

**Apologies—we have added in some more details within our methods and included our data collection instruments as supplements.**

8. The article needs to be a review of grammatical errors.

**Thank you, we have reviewed this manuscript thoroughly.**
