# Supplementary material for: Prevention and management of anaemia in pregnancy: Community perceptions and facility readiness in Ghana and Uganda
Source: PLOS Glob Public Health. 2024 Aug 26;4(8):e0003610. doi: 10.1371/journal.pgph.0003610 (PMC11346734; doi:10.1371/journal.pgph.0003610)
Supplement: Attachment — Submitted filename: Review Comments to the Author.docx [file pgph.0003610.s005.docx]

Review Comments to the Author

Please use the space provided to explain your answers to the questions above. You may also include additional comments for the author, including concerns about dual publication, research ethics, or publication ethics. (Please upload your review as an attachment if it exceeds 20,000 characters) (Limit 2 to 20000 Characters) ?

Has this manuscript never been published in another journal?

**Confidential to Editor**

1. Do you have any potential or perceived competing interests that may influence your review? Please review our [Competing Interests policy](http://journals.plos.org/globalpublichealth/s/competing-interests) and declare any potential interests. If you have no competing interests, please write "I have no competing interests." (Limit 20000 Characters)

I have no competing interests.
